# Supplementary material for: Soil Fungal Pathogens in Pinus pinaster Mature Reforestation: Silvicultural Treatments Effects
Source: Pathogens. 2024 Jul 30;13(8):637. doi: 10.3390/pathogens13080637 (PMC11356966; doi:10.3390/pathogens13080637)
Supplement: Supplementary file 1 [file pathogens-13-00637-s001.zip › pathogens-3039505-supplementary.pdf]

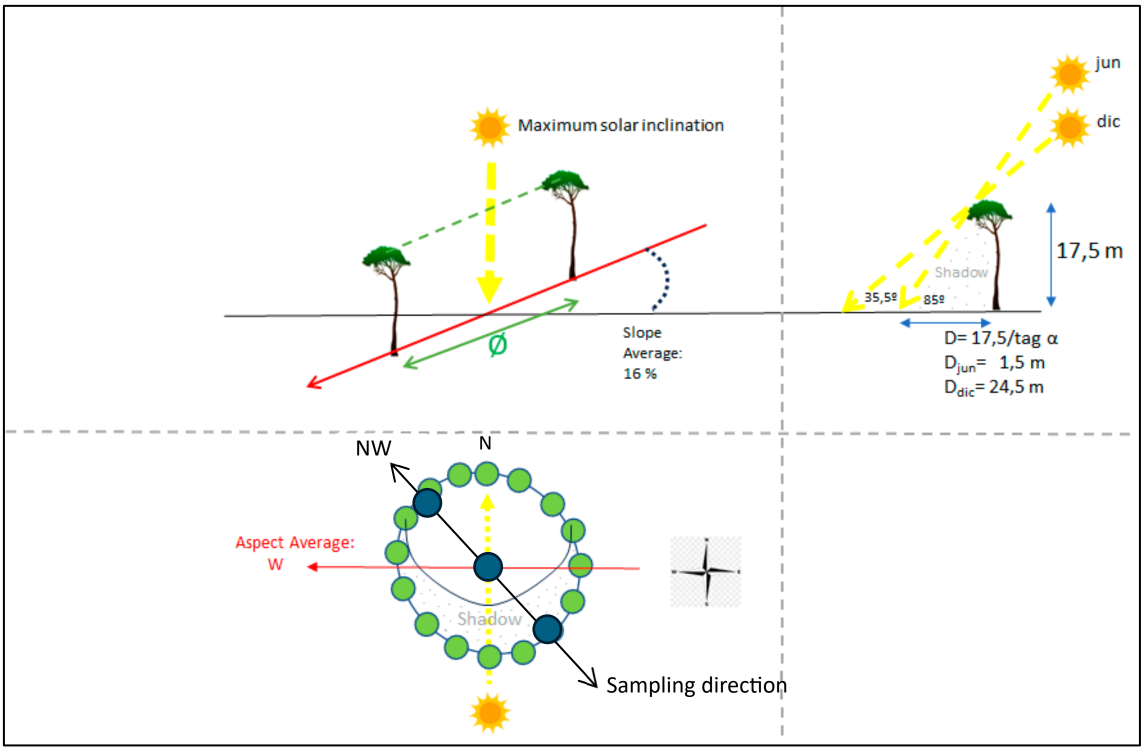

Figure S1. Soil sampling design in Gaps. The effect of insolation without tree cover can mark important changes in the soil environment, with respect to the shaded area. The aspect average of the gaps is W (although it oscillates between N and SW), and the slope is about 16%. In Winter, for the location of the experimental area, and in a situation of maximum solar inclination, most of the area of the 26 m diameter gaps has been shaded by pine canopy, while the 44 m diameter gaps have still had ground area with direct solar incidence.

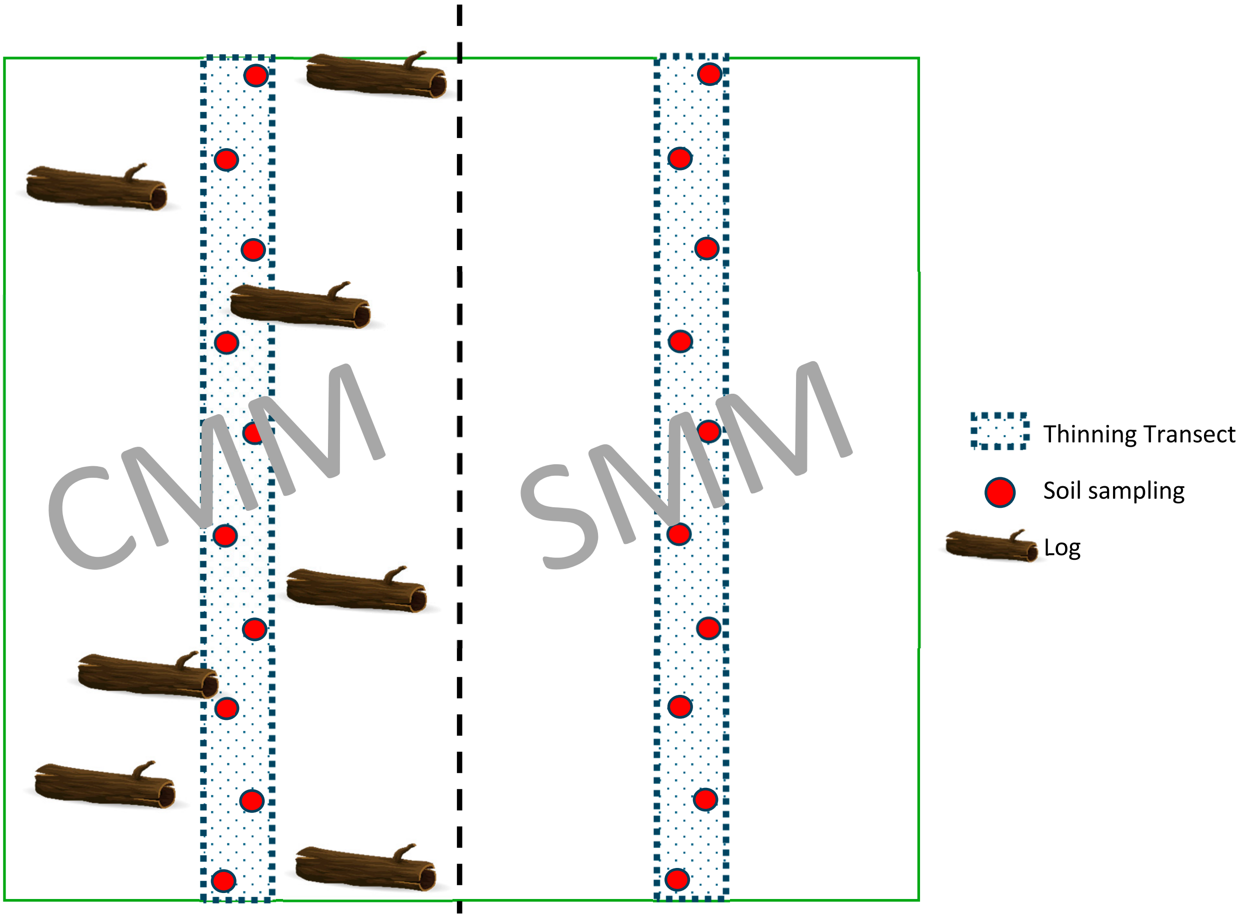

Figgure S2. Soil sampling design in thinning plots. CMM: subplot with coarse woody debris; SMM: subplot without coarse woody debris

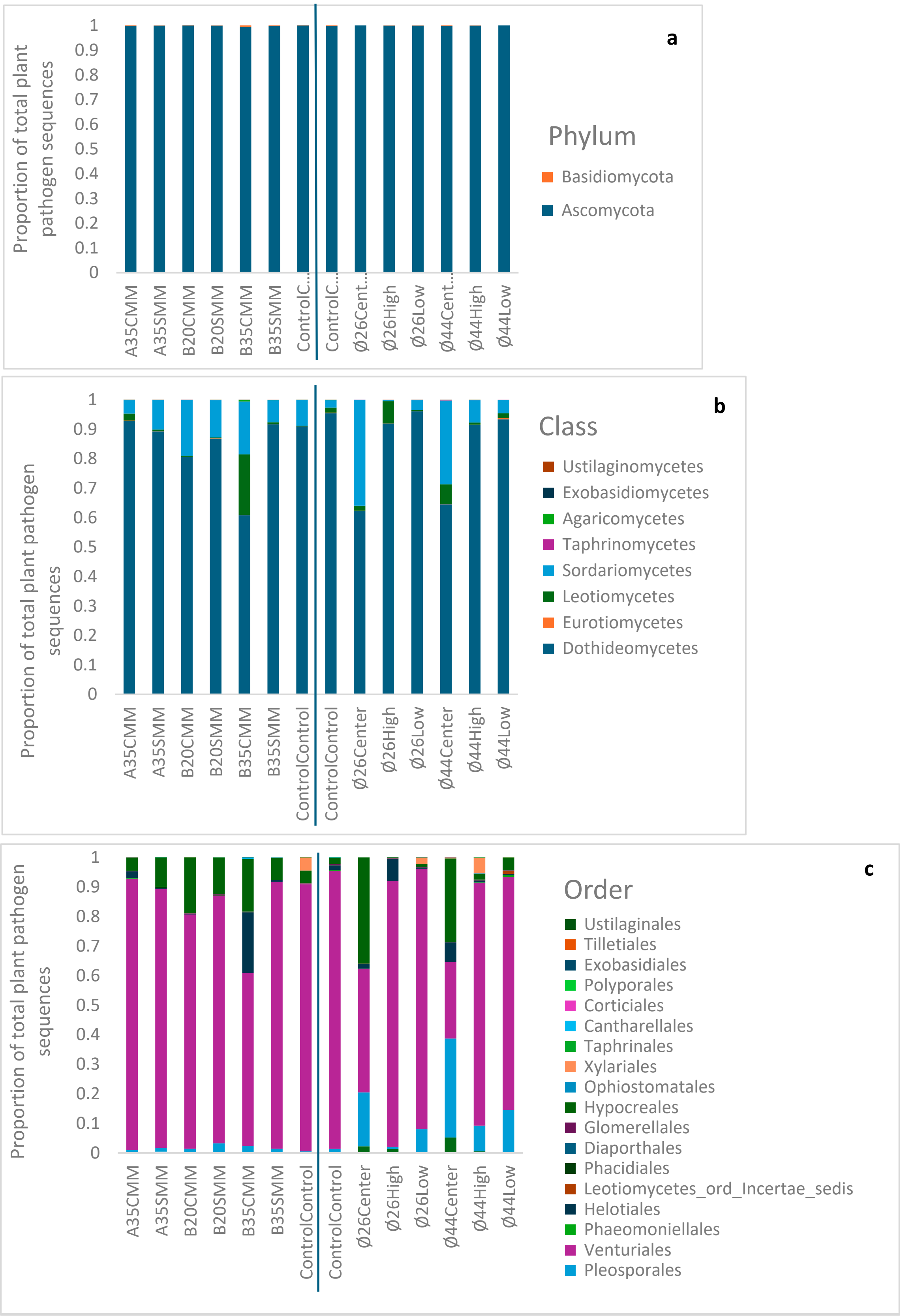

Figure S3. Phylum (a), Class (b) and Order (c) proportion average of total plant pathogen fungi sequences (OTUs) according to silvicultural treatments (thinning or gap), thinning intensity (A35, B35, B20), with (CMM) or without (SMM) coarse woody debris in thinning plots, gap diameters (Ø26 or Ø44) and gap sampling position (High, Center, Low).

Table S1. Average of environmental attributes in sampling sites[1]

NAME	TREATMENT	WEIGHT	BLOCK	POSITION	Aspect (0-360º)	Insolation-Anual (Wh/m²)	Slope (%)	Elevation (m)	Vegetation Cover Index (0-30)
1	Control	Control	1	Control	283	1285621	13	1059	9
2	Gaps	Ø26	1	CENTER	306	1220926	13	1063	0
3	Gaps	Ø26	1	HIGH	304	1220926	13	1065	0
4	Gaps	Ø26	1	LOW	305	1220926	14	1060	2
5	Gaps	Ø44	1	CENTER	333	1170476	15	1062	0
6	Gaps	Ø44	1	HIGH	330	1177883	14	1068	0
7	Gaps	Ø44	1	LOW	332	1155991	16	1056	8
8	Control	Control	2	Control	256	1299422	22	1083	9
9	Gaps	Ø26	2	CENTER	254	1338623	18	1084	0
10	Gaps	Ø26	2	HIGH	254	1331797	17	1085	1
11	Gaps	Ø26	2	LOW	254	1336231	18	1083	3
12	Gaps	Ø44	2	CENTER	274	1275768	15	1108	0
13	Gaps	Ø44	2	HIGH	277	1286903	16	1112	0
14	Gaps	Ø44	2	LOW	285	1275768	15	1105	0
15	Control	Control	3	Control	241	1377789	16	1068	10
16	Gaps	Ø26	3	CENTER	235	1388252	17	1059	0
17	Gaps	Ø26	3	HIGH	232	1394556	18	1059	2
18	Gaps	Ø26	3	LOW	234	1388252	16	1058	1
19	Gaps	Ø44	3	CENTER	247	1349750	17	1031	0
20	Gaps	Ø44	3	HIGH	246	1339687	17	1033	3
21	Gaps	Ø44	3	LOW	250	1347643	18	1029	7
22	Control	Control	4	Control	332	1178803	15	1075	11
23	Thinnings	B20	4	CMM	293	1264018	12	1057	12
24	Thinnings	B20	4	SMM	265	1326627	12	1060	10
25	Thinnings	B35	4	CMM	348	1158557	14	1077	9
26	Thinnings	B35	4	SMM	342	1166338	13	1076	11
27	Thinnings	A35	4	CMM	347	1121313	16	1058	8
28	Thinnings	A35	4	SMM	342	1137646	16	1057	11
29	Control	Control	5	Control	279	1273343	16	1070	10
30	Thinnings	B20	5	CMM	235	1386027	17	1043	6
31	Thinnings	B20	5	SMM	258	1336968	15	1047	8
32	Thinnings	B35	5	CMM	283	1227650	17	1048	8
33	Thinnings	B35	5	SMM	291	1205071	19	1046	8
34	Thinnings	A35	5	CMM	224	1413224	17	1058	7
35	Thinnings	A35	5	SMM	231	1385233	16	1064	8
36	Control	Control	6	Control	267	1335297	10	1059	8
37	Thinnings	B20	6	CMM	308	1207553	14	1051	8
38	Thinnings	B20	6	SMM	314	1245639	13	1049	9
39	Thinnings	B35	6	CMM	243	1359980	10	1049	6
40	Thinnings	B35	6	SMM	251	1355114	10	1052	6
41	Thinnings	A35	6	CMM	292	1208519	17	1058	8
42	Thinnings	A35	6	SMM	296	1206662	16	1056	7

[1] Extracted data using ArcMap 10.8.1©, based on updated digital cartography for public use by the National Geographic Institute of Spain. https://www.scne.es/

Table S2. PERMANOVAs and p-values showing significant differences (red) between fungal communities

Table S2a					
**Source**	**Sum of sqrs**	**df**	**Mean square**	**F**	**p**
Block	1,6556	5	0,33112	0,51893	0,013
Treatment	1,1982	2	0,59911	0,93893	0,0022
Interaction	-10,846	10	-1,0846	-1,6998	1
Residual	15,314	24	0,63808		
Total	7,3213	41			

Table S2b					
**Source**	**Sum of sqrs**	**df**	**Mean square**	**F**	**p**
**Thinning Intensity**	0,65931	3	0,21977	0,53323	0,0264
**CMM/SMM**	0,13676	2	0,068382	0,16592	0,5951
Interaction	-2,5044	6	-0,41741	-1,0128	0,8697
Residual	3,7093	9	0,41214		
Total	2,0009	20			

Table S2c					
**Source**	**Sum of sqrs**	**df**	**Mean square**	**F**	**p**
**Gap Diameter**	0,46985	2	0,23492	0,3359	0,1403
**Sampling Position**	1,4204	3	0,47347	0,67698	0,0032
Interaction	-3,9135	6	-0,65225	-0,93261	0,2155
Residual	6,2944	9	0,69938		
Total	4,2712	20			

Table S2d				
**TREATMENT**	**Thinnings**	**Control**	**Gaps**	
Thinnings		1	0,0003	
Control	1		0,0657	
Gaps	0,0003	0,0657		
**THINNING INTENSITY**	**A35**	**B20**	**B35**	**Control**
A35		1	0,3918	1
B20	1		0,0084	1
B35	0,3918	0,0084		0,072
Control	1	1	0,072	
**SAMPLING POSITION**	**CENTER**	**HIGH**	**LOW**	
CENTER		0,0348	0,0456	
HIGH	0,0348		1	
LOW	0,0456	1		

Table S3. Pearson's correlations between diversity and environmental variables. ***p<0.001; **p<0.01; *p<0.05. Negative correlation (red)

C. Pearson	Taxa_S	Simpson_1-D	Shannon_H	Evenness_e^H/S
Slope				
Alt				
ICV				**
Sun	*			

	Slope	Alt	ICV	Sun	Taxa_S	Simpson_1-D	Shannon_H	Evenness_e^
Slope		0,65651	0,28737	0,32615	0,12528	0,75394	0,73352	0,78512
Alt	0,070671		0,078408	0,47516	0,70052	0,2423	0,24157	0,43535
ICV	-0,16806	-0,27461		0,1881	0,2105	0,12347	0,06119	**0,0023991**
Sun	0,15528	-0,11325	-0,20714		0,02378	0,053677	0,068567	0,36134
Taxa_S	-0,24031	0,061137	0,19727	-0,34834		0,015739	0,010012	0,7622
Simpson_1-D	-0,04984	0,18444	-0,24144	-0,29988	0,37044		1,49E-31	3,43E-13
Shannon_H	-0,054128	0,18471	-0,29136	-0,28378	0,39312	0,98393		2,42E-14
Evenness_e^H	0,04336	0,12364	-0,45611	-0,14447	-0,048117	0,85892	0,87754	

Table S4. Spearman's correlations between main fungal genera and environmental variables.

***p<0.001; **p<0.01; *p<0.05. Positive correlation (Blue) or negative (Red).

C.SPEARMAN Test	Slope	Alt	ICV	Sun
Alternaria			***	
Drechslera		**		*
Gibberella			*	
Ilyonectria	***		**	***
Kabatiella			*	
Mollisia	**			
Nectria	**			**
Ophiosphaerella				*

C.SPEARMAN	Alternaria	Drechslera	Fusarium	Gibberella	Ilyonectria	Kabatiella	Microdoch	Mollisia	Nectria	Ophiospha	Plenodomu	Venturia	Chalastosp	Slope	Alt	ICV	Sun
Alternaria		0,20132	0,053443	2,15E-06	0,91952	0,063041	0,28826	0,9031	0,17445	0,074271	0,90768	0,000181	0,008314	0,90715	0,3602	**0,000777**	0,95937
Drechslera	0,20122		0,59304	0,048809	0,016038	0,037557	0,048922	0,24152	0,000556	0,002556	0,26411	0,040157	0,099586	0,14023	**0,006538**	0,31214	0,017659
Fusarium	0,30016	-0,08488		0,018586	0,2356	0,10457	0,089384	0,77145	0,11333	0,83531	0,10804	0,010942	0,37919	0,84777	0,82229	0,48875	0,60487
Gibberella	0,65832	0,30592	0,36171		0,19973	0,021766	0,41699	0,46688	0,030115	0,095315	0,52543	1,51E-07	0,3728	0,91115	0,9253	0,012894	0,76314
Ilyonectria	0,016075	0,36947	0,18704	0,20192		0,36782	0,023689	0,007384	1,44E-05	0,032202	0,016923	0,016921	0,048557	**0,000334**	0,18301	**0,002957**	**5,09E-05**
Kabatiella	0,2894	-0,32201	0,25398	0,3532	-0,14255		0,4116	0,093375	0,55132	0,36365	0,51209	0,027895	0,66492	0,35312	0,17398	0,02445	0,12209
Microdochium	0,16776	0,30577	-0,26539	0,1286	0,34855	-0,13008		0,029061	0,073414	0,56724	0,39478	0,023851	0,18324	0,52317	0,15119	0,72816	0,051997
Mollisia	0,019369	-0,18473	0,046192	0,11537	-0,40756	0,26226	-0,33704		0,53293	0,59841	0,39318	0,25444	0,24691	**0,007079**	0,28307	0,4871	0,085092
Nectria	0,21357	0,51023	0,24797	0,33499	0,61547	-0,09458	0,27915	-0,09896		0,016168	0,000253	0,003818	0,26721	**0,007534**	0,50695	0,99812	**0,00121**
Ophiosphaerella	0,27835	0,45355	0,033068	0,26077	0,33111	-0,14378	0,090838	-0,08365	0,36905		0,94611	0,13839	0,044284	0,082417	0,48775	0,48956	0,034296
Plenodomus	-0,01845	0,17628	0,25156	0,10077	0,36666	-0,10402	0,13478	-0,13523	0,53614	-0,01075		0,20897	0,63011	0,16614	0,68875	0,66954	0,64107
Venturia	-0,54655	-0,31798	-0,38878	-0,70866	-0,36667	-0,33938	-0,34817	-0,17984	-0,43689	-0,23252	-0,19792		0,13314	0,93465	0,40292	0,17486	0,078608
Chalastospora	0,40201	0,25758	0,13923	0,14109	0,30624	0,068831	0,20939	-0,18267	0,17516	0,31198	-0,07651	-0,23557		0,17952	0,59994	0,80864	0,10245
Slope	0,018556	-0,23147	-0,03054	-0,01775	-0,52725	0,14693	-0,10132	0,40951	-0,40663	-0,27114	-0,21766	0,013046	-0,21114		0,6988	0,23893	0,066958
Alt	0,14481	-0,41316	-0,03572	-0,01492	-0,2095	0,2138	0,22542	0,16955	0,10529	-0,11006	0,063665	-0,13249	-0,0833	-0,06151		0,33323	0,40819
ICV	-0,49862	-0,15978	-0,10981	-0,38062	0,44759	-0,34679	-0,05526	-0,11023	0,000375	-0,10961	0,067825	0,21337	-0,03852	-0,18574	-0,15305		0,15187
Sun	0,008105	-0,36442	-0,08218	0,04792	-0,58302	0,2423	-0,30191	0,26889	-0,48256	-0,32741	-0,07407	0,27444	-0,2555	0,28538	-0,13102	-0,22505	
